# Simultaneous Nanothermometry and Deep‐Tissue Imaging

**DOI:** 10.1002/advs.202000370

**Published:** 2020-04-30

**Authors:** Pascal M. Gschwend, David Niedbalka, Lukas R. H. Gerken, Inge K. Herrmann, Sotiris E. Pratsinis

**Affiliations:** ^1^ Particle Technology Laboratory Institute of Process Engineering Department of Mechanical and Process Engineering ETH Zürich Sonneggstrasse 3 Zurich CH‐8092 Switzerland; ^2^ Institute for Particle Technology Technische Universität Braunschweig Volkmaroder Strasse 5 Braunschweig 38104 Germany; ^3^ Particles‐Biology Interactions Department Materials Meet Life Swiss Federal Laboratories for Materials Science and Technology (Empa) Lerchenfeldstrasse 5 St. Gallen CH‐9014 Switzerland; ^4^ Nanoparticle Systems Engineering Laboratory Institute of Process Engineering Department of Mechanical and Process Engineering ETH Zürich Sonneggstrasse 3 Zurich CH‐8092 Switzerland

**Keywords:** bioimaging, cytocompatibility, flame spray pyrolysis, intravital microscopy, Mn^5+^‐doped Ba_3_(PO_4_)_2_

## Abstract

Bright, stable, and biocompatible fluorescent contrast agents operating in the second biological window (1000–1350 nm) are attractive for imaging of deep‐lying structures (e.g., tumors) within tissues. Ideally, these contrast agents also provide functional insights, such as information on local temperature. Here, water‐dispersible barium phosphate nanoparticles doped with Mn^5+^ are made by scalable, continuous, and sterile flame aerosol technology and explored as fluorescent contrast agents with temperature‐sensitive peak emission in the NIR‐II (1190 nm). Detailed assessment of their stability, toxicity with three representative cell lines (HeLa, THP‐1, NHDF), and deep‐tissue imaging down to about 3 cm are presented. In addition, their high quantum yield (up to 34%) combined with excellent temperature sensitivity paves the way for concurrent deep‐tissue imaging and nanothermometry, with biologically well‐tolerated nanoparticles.

## Graphical abstract

Non‐toxic Mn^5+^‐doped Ba_3_(PO_4_)_2_ nanoparticles smaller than 100 nm are prepared by scalable flame aerosol technology exhibiting ultrabright fluorescence in the preferred spectral region for bioimaging above 1000 nm. They can serve also as nanothermometers within tissues by capitalizing on their temperature‐dependent emission with high quantum yields. The nanoparticle stability, biocompatibility and deep‐tissue imaging down to about 3 cm are elucidated.

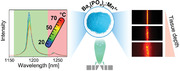

Fluorescence imaging is inherently limited by the depth of light penetration into tissue.^[^ ^1^ ^]^ The emergence of probes and imaging systems operating in the so‐called first biological window^[^ ^2^ ^]^ (650–900 nm) has brought the tissue imaging depth to millimeters. However, over the last couple of years it has become evident that imaging in the second biological window^[^ ^3^ ^]^ (1000–1350 nm) has the prospect to greatly improve imaging quality^[^ ^4^ ^]^ through reduced scattering, absorption, and autofluorescence, enabling the study of centimeter‐deep lying features.^[^ ^5^ ^]^ Despite considerable excitement in the community, actual clinical adoption has been limited due to the lack of suitable fluorescent contrast agents. The currently available probes consist of hardly stable organic dyes such as indocyanine green (ICG),^[^ ^6^ ^]^ heavy‐metal‐based quantum dots (QDs; PbS,^[^ ^7^ ^]^ InAs^[^ ^8^ ^]^) raising toxicity concerns, and lanthanide‐doped materials suffering from low absorption cross‐sections.^[^ ^9^ ^]^ Therefore, there is need for alternative nanophosphors that exhibit strong stability, high brightness, and low (if any) toxicity.^[^ ^10^ ^]^

Toward this goal, it has been shown that many fluorescent agents (e.g., LaF_3_:Nd^[^ ^11^ ^]^ or PbS/CdS/ZnS QDs^[^ ^12^ ^]^) are highly sensitive to temperature changes in their surroundings, and could therefore be used for thermal sensing. Such (sub‐)nanoscale probes inherently represent the only option to measure local temperature within tissue in a contactless manner in real‐time.^[^ ^13^ ^]^ Local temperature sensing has important applications, e.g., for cancer diagnosis and therapy. However, the noncontact temperature measurements within biological tissues are challenging^[^ ^14^ ^]^ due to strong light absorption and highly wavelength‐dependent optical properties. Thus, careful selection of operation wavelengths and read‐out strategy is crucial. Fluorescent nanothermometers (e.g., LaF_3_:Nd) can be employed to monitor and control the local temperature during hyperthermia treatment of cancer,^[^ ^11^ ^]^ thereby reducing the potential for unnecessary damage to surrounding healthy tissue. However, the LaF_3_:Nd with sensitivity of 0.25% K^−1^ operates in the NIR‐I, and hence application is limited to superficial features due to the limited tissue penetration. Since temperature affects the dynamics and structure in biological systems,^[^ ^15^ ^]^ localized temperature measurements can provide insight into previous inaccessible processes. For example, Santos et al.^[^ ^16^ ^]^ have recently demonstrated the diagnosis of tumors 6 days earlier than by visual inspection, by characterizing the thermal relaxation dynamics of the tissue by nanothermometry with Ag‐containing QDs.

Most of the reported fluorescent thermometers are based on lanthanides due to their rich spectroscopic features. In contrast, reports on nanoscale fluorescent agents capable of thermal sensing based on transition metal ions are sparse, despite their well‐known susceptibility to thermal quenching. While several nanothermometry probes have been proposed for imaging in the first biological window (Cr^3+^,^[^ ^17^ ^]^ Co^2+^,^[^ ^18^ ^]^ Mn^3+^/Mn^4+^,^[^ ^19^ ^]^ Ti^3+^/Ti^4+^,^[^ ^20^ ^]^ and V^3+^/V^5+[^ ^21^ ^]^), only a Ni^2+^‐based probe has been reported so far for the second biological window with its superior tissue imaging depth.^[^ ^22^ ^]^ Most of the aforementioned studies have focused solely on the thermometric performance. Few reports have considered other application‐dependent constraints related to particle size, brightness, stability, or biocompatibility. Here, we report on biocompatible Mn^5+^‐doped barium phosphate nanoparticles (*d* < 100 nm) for bifunctional temperature‐sensitive deep‐tissue imaging. Even though Mn^5+^‐doped Ba_3_(PO_4_)_2_ particles have been suggested as fluorescent agents,^[^ ^23^ ^]^ many parameters crucial to bioimaging (such as particle size, cytocompatibility, or colloidal stability) had not been evaluated.

Mn^5+^‐doped barium phosphate nanoparticles were prepared by flame aerosol technology^[^ ^24^ ^]^ (Figure S1a, Supporting Information) and annealed at 800 °C in air for 2 h. To optimize the fluorescence intensity, first the Ba to P molar ratio was systematically varied from 1.45 to 1.75, leading to the formation of Mn‐doped Ba_2_P_2_O_7_, Ba_3_(PO_4_)_2_, and Ba_5_(PO_4_)_3_OH (Figure S1b, Supporting Information). **Figure** **1**a shows the X‐ray diffraction (XRD) pattern of a powder (Ba/(P+Mn) = 1.55) that consists of 75% Ba_3_(PO_4_)_2_ (triangles) and 25% Ba_5_(PO_4_)_3_OH (circles). Of these, Ba_5_(PO_4_)_3_OH could most effectively stabilize Mn in the 5+ valence state, as evidenced by the blue coloration^[^ ^25^ ^]^ (Figure S2a–h, Supporting Information) and stronger absorption (Figure 1c, circles, and Figure S2i, Supporting Information) of the powder, in analogy to calcium phosphate,^[^ ^26^ ^]^ as well as by Raman spectroscopy (Figure S3, Supporting Information). This was further supported by elemental analysis, where the Mn content (all valence states) was comparable (± 6% relative deviation) for all hosts (Figure S2j, Supporting Information). While absorption was lower for Ba_3_(PO_4_)_2_, the fluorescence intensity was highest. The maximum intensity was observed at a nonstoichiometric ratio of 1.55 with the formula Ba_3_(P_0.99_Mn_0.01_O_4_)_1.935_ (Figure 1c, triangles), which from now on is labeled as BaPOMn.

**Figure 1:**
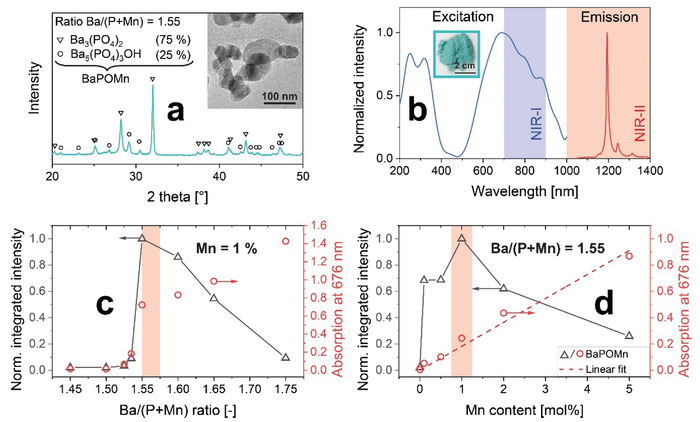
a) XRD pattern and transmission electron microscope (TEM) image (inset) of air‐annealed (2 h) BaPOMn consisting of 3:1 Ba_3_(PO_4_)_2_ (triangles) and Ba_5_(PO_4_)_3_OH (circles). b) Excitation and emission spectra of these particles (inset: powder image) where the first (NIR‐I) and second (NIR‐II) biological windows are indicated (shaded areas). Fluorescence (triangles) and absorption (circles) intensities as a function of c) Ba/(P+Mn) ratio at 1% Mn content, and d) Mn content at Ba/(P+Mn) = 1.55.

The excitation and emission spectra of BaPOMn (Figure 1b) are characterized by a broad excitation peak in the NIR‐I (blue‐shaded area) and sharp emission line at 1190 nm in the NIR‐II (red‐shaded area), making them most attractive for deep‐tissue imaging. These spectral properties are characteristic for Mn^5+^ in a strong crystal field, which was calculated^[^ ^27^ ^]^ as *Dq*/*B* = 3.22, in agreement with Laha et al.^[^ ^28^ ^]^ Following the matrix optimization, the Mn doping concentration was optimized and its fluorescence (Figure 1d, triangles) peaked at 1 mol% in agreement with literature,^[^ ^23^, ^29^ ^]^ while the absorption intensity increased linearly with Mn content (Figure 1d, circles). The actual Mn contents determined by inductively coupled plasma‐optical emission spectrometry (ICP‐OES) were in excellent agreement with the nominal ones (*R* ^2^ = 0.99, Figure S4a, Supporting Information). The optimization of Mn content resulted in remarkably high quantum yields (QYs, determined by an absolute method using an integrating sphere^[^ ^30^ ^]^) reaching up to 34.4% for the lowest doping concentration (0.1 mol%) and 22% for the brightest probe (1 mol%) (Figure S4b, Supporting Information). Furthermore, larger, rather aggregated BaPOMn particles (*d*_BET_ = 310 nm, *d*_XRD_ = 126 nm) obtained by annealing at higher temperatures (1000 °C for 2 h in air) increased the QY (26.8%), most likely due to the reduction of surface defects by annealing.^[^ ^31^ ^]^ The QY of dispersions of BaPOMn (1 mol%, annealed at 800 °C in air for 2 h) was 5.4 ± 1.6% in water and 7.1 ± 1.3 in phosphate‐buffered saline (PBS). These QYs are among the highest reported in this spectral region, e.g., single‐walled carbon nanotube (0.1–1%),^[^ ^32^ ^]^ Ag_2_S QD (15.5%),^[^ ^33^ ^]^ or ICG (13.2%, main peak in NIR‐I).^[^ ^34^ ^]^ It is worth pointing out that most of these QY are determined relative to reference dyes (such as IR‐26) whose reported QY varies by an order of magnitude.^[^ ^35^ ^]^

Particle size is important for biomedical imaging affecting biodistribution and tumor targeting efficiency.^[^ ^36^ ^]^ The mean crystal size (*d*_XRD_) of annealed particles (Figure 1a) was 44 nm, smaller than the primary particle size by N_2_‐adsorption (*d*_BET_ = 69 nm) or by microscopy (*d*_TEM_ = 64 nm, Figure S5a and elemental mapping in Figure S6, Supporting Information). The deviation is attributed to the formation of sinter necks (e.g., aggregation) during annealing (inset Figure 1a). However, large aggregates can be removed by centrifugation of aqueous dispersions (Figure S5b, Supporting Information), resulting in nanoparticle dispersions with hydrodynamic sizes around 100 nm, which is well in line with sizes desired for tumor‐targeting.^[^ ^37^ ^]^ The nanoparticle colloidal stability after adsorption of human serum albumin was excellent for, at least, 72 h (Figure S7, Supporting Information) in five biologically relevant media: H_2_O, PBS, Dulbecco's Modified Eagle's Medium (DMEM), physiological saline (0.154 m NaCl), and serum. The nanoparticles’ emission characteristics were not affected by adsorbed proteins (Figure S8, Supporting Information) and they also retained their functionality in a broad pH range (Figure S9a, Supporting Information). Furthermore, these nanophosphors are not affected by photobleaching which is common for organic dyes^[^ ^6^ ^]^ and QDs:^[^ ^38^ ^]^ The fluorescence intensity of nanophosphors remained unaltered for, at least, 1 h under continuous laser excitation. In contrast, commercially available PbS‐CdS QD and ICG reduced their emission intensity by 50% and 100%, respectively, within the first 10 min already (Figure S9b, Supporting Information).

The cytocompatibility of these Mn‐doped barium phosphate particles was assessed with three cell lines, namely, the widely used carcinoma HeLa cell line, human monocytes (THP‐1), and normal human dermal fibroblasts (NHDF). **Figure** **2**a–c (and Figure S10, Supporting Information) shows the viability of these cells after 24 h of incubation at different nanoparticle concentrations (blue columns) given by an adenosine triphosphate quantifying cell viability assay. The BaPOMn nanoparticles caused no significant reduction in cell viability for all three cell lines. Higher concentrations were not measured due to the limited feasibility of these assays at ultra‐high concentrations,^[^ ^39^ ^]^ even though no interferences could be detected up to 1000 µg mL^−1^.

**Figure 2:**
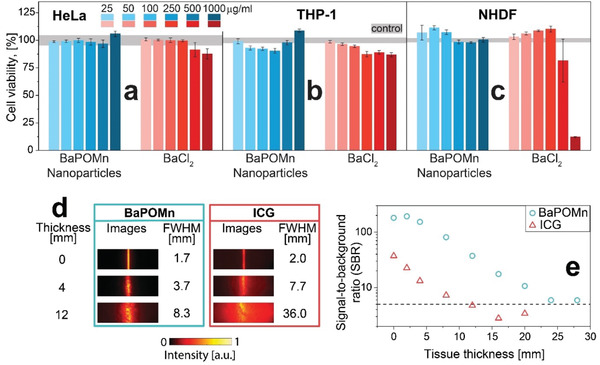
Cytocompatibility of BaPOMn nanoparticles and BaCl_2_ salt at different concentrations (25–1000 µg mL^−1^) with a) HeLa cells, b) THP‐1 monocytes, and c) NHDF cells. The gray band denotes the standard deviation of the control cells. Deep‐tissue imaging with the organic dye ICG and BaPOMn nanoparticles in 2 mm thick capillaries under tissue of different thicknesses. d) Fluorescence images and FWHM at the corresponding tissue thickness. e) SBR as a function of tissue thickness. Dashed line indicates the Rose criterion^[^ ^41^ ^]^ with SBR of 5.

To assess the influence of potential release of Ba^2+^ ions on cell viability, water soluble BaCl_2_ was also tested (red columns) under the same conditions. The viability of HeLa and THP‐1 monocytes is hardly affected by Ba^2+^ and only decreases for concentrations >250 µg mL^−1^, whereas the NHDF cell viability shows a strong reduction at 500–1000 µg mL^−1^. The LD50 of BaCl_2_ for NHDF is 551 ± 21 µg mL^−1^, similar to those for L929 fibroblasts (440 µg mL^−1^).^[^ ^40^ ^]^ These results were in agreement with the release of lactate dehydrogenase by these cells (Figure S11, Supporting Information). Furthermore, the particles were also well tolerated by cells during longer exposures (e.g., 48 h, Figure S12, Supporting Information), showing no significant difference to the results after 24 h incubation. Given the high cell viability even after prolonged particle exposure, a major activation of the apoptosis pathways is not expected. This was confirmed by an apoptosis detection assay (Figure S13, Supporting Information).

The fraction of leached barium from barium phosphate nanoparticles (500 µg mL^−1^) after 1 day in H_2_O, PBS, DMEM, and physiological saline was quantified by ICP‐OES (Figure S14, Supporting Information), and reached 2% (relative to total dose) in cell culture medium. Thus, very high nanoparticle concentrations (≈25 mg mL^−1^) would be required to induce a 50% cell viability reduction only due to Ba^2+^ ions.

To assess the potential for in vivo deep‐tissue imaging, BaPOMn nanoparticles with relatively high emitter concentration (*c* = 1 g L^−1^), common for intratumoral administration,^[^ ^42^ ^]^ were filled into small capillaries (diameter = 2 mm, length = 4–6 cm) and covered by layers of turkey muscle tissue. For comparison, the same experiment was conducted with commercially available organic dye ICG (*c* = 0.01 g L^−1^, concentration with brightest emission^[^ ^43^ ^]^). The fluorescent agents were illuminated under harmless laser intensities (*λ* = 808 nm, 0.19 W cm^−2^) and the emitted signal was filtered with a 850 nm longpass and collected with a near‐infrared (NIR) camera, where the exposure time was adapted to reach a signal close to saturation (1–50 ms). Figure 2d shows the fluorescence images of nanoparticles (left) and ICG (right) under increasingly thick layers of tissue (see Figure S15a in the Supporting Information for all thicknesses).

The performance to resolve deeper lying structures was quantified by the full‐width at half‐maximum (FWHM) of the capillary (Figure 2d and Figure S15b, Supporting Information). For BaPOMn, the FWHM was increased from 1.7 to 8.3 mm when covered with 12 mm thick tissue. In contrast, the FWHM of ICG reached 36 mm at the same thickness. This can be attributed to stronger scattering in the NIR‐I, showing the benefit of operating in the NIR‐II. Similarly, the signal intensity as a function of tissue thickness was quantified in Figure 2e by the signal‐to‐background ratio (SBR). The ICG (triangles) can be detected up to a tissue thickness of 12 mm. With thicker tissues, its SBR drops below 5 (dashed line), which is the minimum required to distinguish image features with 100% certainty according to Rose criterion.^[^ ^41^ ^]^ In contrast, nanoparticles can be detected up to 28 mm while maintaining an SBR above 5, in line with previous theoretical^[^ ^44^ ^]^ and experimental reports.^[^ ^45^ ^]^ This can be explained by the reduced absorption in the NIR‐II and their higher brightness. However, as the emission intensity scales linearly with particle concentration (Figure S16, Supporting Information), this imaging performance decreases for lower local concentrations.

The generated signal, however, carries more information than just intensity! Transition metal ions are very sensitive to the surrounding temperature.^[^ ^46^ ^]^ Therefore, we investigated here, for the first time to our knowledge, the use of Mn^5+^ for nanothermometry. **Figure** **3**a shows the spectrum of aqueous nanoparticle dispersion from 10 to 70 °C, while powder spectra from 25 to 200 °C are shown in Figure S17a in the Supporting Information. Several features of the spectra change with temperature and can therefore be used for thermal read‐out.

**Figure 3:**
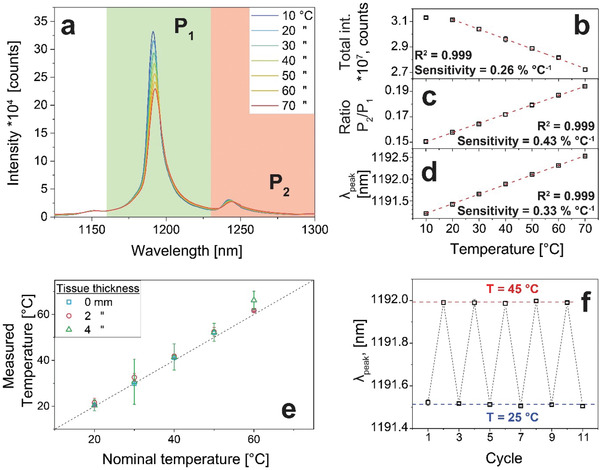
Nanothermometry of annealed BaPOMn: a) temperature‐dependent fluorescence spectra between 10 and 70 °C. Three parameters can be used for thermal sensing: b) the total integrated intensity, c) the ratio between the two peaks *P*_2_/*P*_1_, and d) the peak position. e) Temperature measurements through tissue of different thickness. f) Repeatability over 10 cycles between 25 and 45 °C.

The total integrated intensity (Figure 3b) decreases by thermal quenching,^[^ ^47^ ^]^ which can be fitted linearly from 20 to 70 °C. While this approach is the simplest in terms of experimental implementation, it is only feasible in very stable conditions. Since it lacks reference, any change in local particle concentration or tissue absorption can distort the temperature readout.

As a second readout, the ratio between the main emission line (*P*_1_, 1160–1230 nm) and the vibronic sideband^[^ ^47^ ^]^ (*P*_2_, 1230–1300 nm) was used. Since the sideband is hardly affected by thermal quenching (Figure S17b, Supporting Information), the ratio *P*_2_/*P*_1_ increases linearly with temperature (Figure 3c). While such ratiometric temperature sensing is independent of particle concentration,^[^ ^14^ ^]^ it still requires the absorption spectrum of the surroundings to be relatively constant to operate at varying depths.

Third and finally, also the peak position of the main emission line (*λ*_peak_) red‐shifts with increasing temperature, from 1191.1 to 1192.5 nm between 10 and 70 °C (0.022 nm °C^−1^, Figure 3d). The peak position does not depend on local particle concentrations and is not influenced by the absorption of the surroundings, therefore making it a very robust anchor for deep‐tissue thermal sensing.

The response to temperature changes has been linearly fitted with excellent *R* ^2^ = 0.999 for all of them and was quantified in terms of relative thermal sensitivity, which were 0.26, 0.43, and 0.33% °C^−1^ for the three approaches. While these values are not among the highest reported (Table S1, Supporting Information), they arise from a simple singly doped material, our BaPOMn. Similarly, also the thermometric performance of Ba_5_(P_0.99_Mn_0.01_O_4_)_3_OH particles (having Ba/(P+Mn) = 1.75) was investigated, resulting in even higher sensitivities up to 0.83% °C^−1^ (Figure S18, Supporting Information). However, due to the higher emission intensity of BaPOMn (Figure 1c, Ba/(P+Mn) = 1.55), we focused on the latter. Furthermore, the repeatability over 10 cycles was excellent (Figure 3f), and the temperature uncertainty (or resolution) was below 1 °C (Figure S17c, Supporting Information).

Finally, the ability to measure the temperature through biological tissues was investigated by placing tissues of different thickness (0–4 mm) around a thermally controlled cuvette filled with an aqueous nanoparticle suspension (Figure S17d, Supporting Information). The temperature of the cuvette was varied from 20 to 60 °C and the peak position was used to determine the temperature (Figure 3e). The temperatures determined by the nanoparticles are in excellent agreement with the nominal ones. While the standard deviation increases with tissue thickness due to lower signal intensity caused by tissue absorption, the thermal imaging depth limit of 4 mm is on par with Ag/Ag_2_S^[^ ^48^ ^]^ or BiVO_4_:Nd,^[^ ^14^ ^]^ and above Y_2_O_3_:Nd^[^ ^49^ ^]^ or SrF_2_:Nd,Gd^[^ ^50^ ^]^ and perfectly feasible for intravital microscopy applications in small animals.

In conclusion, Mn^5+^‐doped barium phosphate nanoparticles with sizes below 100 nm can serve as cytocompatible fluorescent contrast agents in the NIR‐II for deep‐tissue imaging down to 2.8 cm. These nanoparticles made by scalable and sterile flame spray pyrolysis exhibit ultra‐bright and stable emission in the NIR‐II window with high temperature sensitivity as shown with animal tissue of increasing thickness.

## Conflict of Interest

The authors declare no conflict of interest.

## Supporting information

Supporting Information
