# Supplementary material for: Simultaneous Nanothermometry and Deep‐Tissue Imaging
Source: Adv Sci (Weinh). 2020 Apr 30;7(12):2000370. doi: 10.1002/advs.202000370 (PMC7312269; doi:10.1002/advs.202000370)
Supplement: Supplementary file 1 — Supporting Information [file ADVS-7-2000370-s001.pdf]

ELECTRONIC SUPPORTING INFORMATION

Simultaneous Nanothermometry and Deep-tissue Imaging

Pascal M. Gschwend[1], David Niedbalka[2,1], Lukas R.H. Gerken[3,4], Inge K. Herrmann[3,4] and Sotiris E. Pratsinis[1].*

Materials: Barium acetate (Sigma-Aldrich, purity 99 %), manganese(II) 2-ethylhexanoate (Mn-2-EH, Alfa Aesar, 40% w/w in mineral spirits, 6% Mn), tributyl phosphate (TBP, Sigma-Aldrich, purity 97 %) and 2-ethylhexanoic acid (2-EHA, Sigma-Aldrich, purity 99%). Indocyanine green (ICG, Cardiogreen for microscopy) and PbS/CdS quantum dots (QDs, water soluble, core diameter = 3.1 nm, shell thickness = 0.1 nm, diol coating, 3.857 g/L) were purchased from Fluka Analytical and Nano Optical Materials, respectively.

Synthesis: Barium phosphates were prepared by flame-spray pyrolysis.[22] In general, the spray nozzle was fed by 5 ml/min precursor solution and dispersed by 5 L/min oxygen (PanGAS, purity >99%) with a pressure drop of 1.5 bar. The spray flame was sustained by a ring-shaped pilot flame of oxygen/methane fed at a ratio of 3.2/1.5 L/min. Different Mn-doped barium phosphates according to the stoichiometric equation $Ba_3(P_{1-x}Mn_xO_4)_y$ with x=0-0.05 (0-5 mol%) and various Ba/(P+Mn) ratios with y=2.069-1.714 (Ba/(P+Mn)=1.45-1.75) were prepared. Precursors were prepared based on a 0.4 M metal precursor concentration. Barium acetate was dissolved in 2-EHA for at least 12 h at 130 °C under reflux and stirring and served as stock solution. Firstly, tributyl phosphate (TBP) and, secondly, Mn-2-EH, were added in stochiometric amounts and stirred for another 30 min before spraying as precursor solution. For each batch, roughly 70 ml were sprayed. As-prepared particles were collected on a glass microfiber filter (Whatman GF) by a gas pump (Busch Mink MM 1202 AV). The burner to filter distance was kept at 60 cm, maintaining the filter temperature below 200 °C. As-prepared powders were annealed in air at different temperatures ranging from 600-1000 °C for 2 h in 20 ml alumina crucibles.

Characterization: Powder X-ray diffraction (XRD) patterns were collected on a Bruker AXS D8 ADVANCE diffractometer (40 kV, 30 mA, Cu Kα radiation) at 2θ = 15-70° with a step size of 0.0145°. The pattern and crystal sizes were evaluated with the software TOPAS 4 (Bruker) and Rietveld fundamental parameter refinement. The specific surface area (SSA) of

the particles was determined by Brunauer-Emmer-Teller (BET) method at 77 K (Micrometrics, TriStar 2 Plus). Powders were degassed for at least 1 hour at 150 °C before each measurement. Particle sizes (d_{BET}) were determined by:

$$d_{BET} = \frac{6}{\rho \cdot SSA}$$

where ρ stands for the density of the material. The following densities were used to calculate d_{BET}: 5.254 g/cm^3 for $Ba_3(PO_4)_2$; 4.73 g/cm^3 for $Ba_5(PO_4)_3OH$ and 4.11 g/m^3 for σ-$Ba_2P_2O_7$. For mixed phases, an average density was calculated based on the relative mass fractions obtained by fitting of the XRD patterns.

Primary particle size and morphology were analyzed by (scanning) transmission electron microscopy ((S)TEM, Talos F200X, Thermo Fisher Scientific, Eindhoven, The Netherlands) at an acceleration voltage of 200 kV. For elemental mapping STEM was combined with energy-dispersive X-ray spectroscopy (EDXS) using four attached silicon drift detectors. TEM images were evaluated by drawing two perpendicular lines for each particle. Hydrodynamic particle sizes were measured by dynamic light scattering (DLS, Zetasizer LS, Malvern). Suspensions were prepared by dispersing nanoparticles and human serum albumin (HSA, Sigma-Aldrich, > 97 %) (Gschwend, et al., 2019) at equal mass concentration by ultrasonication (cup-horn system, Vibracell, 90 % amplitude, 28 s ON, 2 s OFF), 10 minutes total) in deionized water.

Diffuse reflectance spectra of powdered samples were collected by UV-Vis spectroscopy (UV-Vis V-770 Spectrophotometer, Jasco) with an integrating sphere in the range of 300 – 1250 nm. Absorption intensities were calculated based on the diffuse reflectance spectra using the Kubelka-Munk (Kubelka and Munk, 1931) function:

$$F(R) = \frac{(1-R)^2}{2R}$$

where $F(R)$ stands for the absorption as function of absolute reflection R. Before the measurements, the reference was measured and compared to the background to decrease uncertainties.

NIR-II fluorescence emission spectra of dry powders were measured by a Spectrofluorometer FS5 (Edingburgh Instruments), where the spectral sensitivity of the detectors was accounted for. Materials were always excited with an 808 nm laser (PSU-3-LED, Changchun New Industries Optoelectronics tech. co., LTD) and an intensity of 1.57 A to a corresponding value of 1.176 W unless mentioned otherwise. The photoluminescence quantum yield (QY), as the ratio of emitted to absorbed number of photons, was measured for dry powders by the same spectrofluorometer equipped with an integrating sphere (150 mm diameter) and a laser intensity of 0.49 A (0.04 W). The absorption (difference between scattered light by reference and sample) and emission range were scanned from 775 to 830 and 1050 to 1400 nm, respectively. The QY was calculated by:[26]

$$\phi = \frac{N_{em}(\lambda_{ex})}{N_{abs}(\lambda_{ex})} = \frac{\int_{1050nm}^{1400nm} I_{sample} - \int_{1050nm}^{1400nm} I_{reference}}{\int_{775nm}^{830nm} I_{reference} - \int_{775nm}^{830nm} I_{sample}}$$

where N_{em} is the number of emitted photons, N_{abs} the number of absorbed photons, λ_{ex} the excitation wavelength and I corresponds to the intensities. As reference material, a highly scattering $BaSO_4$ plug was used (Edinburgh Instruments). In contrast to relative quantum yield measurements, this method avoids the need for reference materials that are scarce[29] in the NIR, as well as not always reliable (Semonin, et al., 2010). For QY measurements of suspensions, particles were dispersed in water or PBS (2 mg/ml) using ultrasonication

together with HSA (2 mg/ml). The suspensions were placed within the same integrating sphere and excited with a laser (808 nm, 1mW). As a reference material, an undoped $Ba_3(PO_4)_2$ sample was employed with similar scattering properties (Boyer and van Veggel, 2010).

NIR-II emission intensities of water-dispersed particles were measured by the same equipment with a different set up containing a temperature-controlled cuvette holder. Prior to measurements, particles were dispersed by ultrasonication (a cup-horn system (Vibracell), 90 % amplitude, 28 s ON, 2 s OFF) in deionized water with HSA. Dispersions were always magnetically stirred at 500 or 1000 rpm and maintained at 20 °C in 3 ml polystyrene cuvettes during the measurements.

The composition of nanoparticles was determined by ICP-OES (Agilent 5110). The particles were dissolved by slowly adding roughly 2 ml of HNO_3 (Fisher Scientific, 65 %) followed by 1 ml H_2O_2 (Alfa Aesar, 35%). In case the nanoparticles did not dissolve entirely, additional amount (0.1-1 ml) of H_2O_2 was added and the solution was ultrasonicated for 10 min. The solution was then diluted with ultrapure water (Milli-Q, IP-7000, 18.2MΩ・cm at 25 °C, 5.2 ppb total organic content) to a 5 – 10 mg/L end concentration.

Raman spectroscopy was performed using a confocal Raman microscope (alpha 300R, WITec, Ulm, Germany). Spectra were collected using a 532 nm laser and a 50x (0.55 NA) LD objective (Zeiss LD EC Epiplan-Neofluar Dic 50x / 0.55). A 600 lines per mm spectral grating was used (UHTS 300, WITec, Ulm, Germany). The laser power was set to 10 mW. Integration time was 2 s and the number of accumulations was 10.

Cell experiments: Human cervical carcinoma (HeLa) cells (ATCC CCL-2) were cultured in Minimum Essential Medium (MEM) containing 10 % fetal calf serum (FCS), 1 % of L-Glutamine, penicillin-streptomycin-neomycin (PSN) and non-essential amino acids

(NEAA), and 1 mM sodium pyruvate. Normal human dermal fibroblasts (NHDF) cells were cultured in Dulbecco's Modified Eagle Medium (DMEM) with 10% FCS and 1% PSN, and human monocytic (THP-1) cells in Roswell Park Memorial Institute (RPMI) 1640 Medium containing 10% FCS. All cells were cultured at 37 °C in a humidified atmosphere containing 5 % CO_2. The cells were sub-cultured after growing to 60-80 % confluency. To evaluate the cytotoxicity of nanoparticles, NP stock suspensions were prepared in ddH_2O at 5 mg/mL NP concentration with the addition of HSA (at the same weight concentration) and subsequent 2 min tip sonication. 10.000 Hela or 8.000 NHDF cells were seeded in a sterile black 96 well plate with transparent bottom in a full cell culture medium (80 µL) and left to attach overnight. THP-1 cells were seeded at 80.000 cells per well in full culture medium (160 µL) on the day of NP incubation. First, the corresponding amounts of ddH_2O, then freshly prepared NP suspension were added at the desired amounts to the cells. The resulting final particle concentrations were ranging from 0 to 0.025, 0.05, 0.1, 0.25, 0.5 and 1 mg/mL. The total liquid volume per well was 100 µL (NHDF, HeLa) and 200 µL (THP-1). The total ddH_2O content was 20 % in each well and ddH_2O was used as a cytotoxic negative control while Lysis solution (Promega, G182A) as a cytotoxic positive control. After NP addition, cells were incubated for 24 or 48 hours at 37 °C under humidified atmosphere containing 5 % CO_2. After incubation, 50 µL of supernatant of each well was transferred to a new 96 well plate and lactate dehydrogenase (LDH) was measured as specified by the manufacturer. In short, 50 µL of LDH substrate (CytoTox 96® Non-Radioactive Cytotoxicity Assay, Promega, G1780) was added and the plates were incubated in the dark for 30 minutes at room temperature. Absorbance at 490 nm was measured using a Mithras LB 943 Multimode Plate Reader. The remaining cell supernatant was discarded and 50 µl of fresh full cell culture medium was added to each well (NHDF, HeLa), or 100 µL supernatant was left in each well (THP-1). To this, CellTiter-Glo® Reagent (CellTiter-Glo® Luminescent Cell Viability Assay, Promega, G7571) was added (1:1 volume) and after 15 min incubation on the shaker in the

dark, luminescence was recorded with the plate reader. Analysis of the reader values was done by background subtraction and normalizing to the corresponding positive control. For apoptosis measurements, cells were seeded and incubated with BaPOMn nanoparticles and $BaCl_2$ salt using the above described conditions. Staurosporine (1 mM in DMSO) was diluted to a final concentration of 1 µM in cell culture media and served as positive control. An apoptosis detection assay based on Annexin V (Apoptosis Detection Assay, Promega) was used according to the manufacturer's protocol. Briefly, signs of apoptosis were detected based on two annexin V fusion proteins (Annexin V-LgBiT and Annexin V-SmViT) containing complementary subunits of NanoBiT Luciferase. Luminescence was monitored using a Mithras plate reader 6 hours post-exposure.

Photostability was measured by photoluminescence spectroscopy with the above mentioned set up for suspensions containing a temperature-controlled holder with magnetic stirring. Roughly 2 ml of BaPOMn nanoparticles, commercially available ICG and PbS/CdS QDs with a 0.01 g/L concentration were dispersed in water. Prior to measurements, BaPOMn nanoparticles were ultrasonicated with HSA (1:1 wt ratio) as described above. Samples were irradiated for 1 h by a laser (λ_{ex} = 808 nm, power density of 8.32 W/m^2) and photoluminescence intensities at the respective peak wavelengths were measured every 5 s.

The chemical stability of BaPOMn nanoparticles was measured by photoluminescence spectroscopy by dispersing 0.5 g/L (1:1 wt ratio HSA, prepared as described above) in aqueous solutions with pH of 2 to 10. The pH of stock solutions was adjusted prior to adding the nanoparticles using 0.1 M NaOH and HCl (Sigma-Aldrich). Samples were shaken at 500 rpm and kept at 37 °C to imitate body core temperature. Fluorescence spectra in solution were recorded as mentioned above after 1 and 24 h to compare their intensities.

The colloidal stability of BaPOMn nanoparticles was measured by DLS. They were dispersed in water with HSA as mentioned above and centrifuged at 3000 rpm (Centrifuge

5430, Eppendorf). The supernatant was dispersed in deionized water, Dulbecco's phosphate-buffered saline (PBS, modified, without calcium chloride and magnesium chloride), Dulbecco's modified eagle medium – high glucose (DMEM), 0.154 M sodium chloride in water (NaCl, BioXtra, purity ≥ 99.5 %), and serum in a 1 to 3 ratio (all acquired from Sigma-Aldrich). The particle size was measured every 24 h for three consecutive days and the mean particle size of the number distribution is reported together with the standard deviation.

The BaPOMn nanoparticles were sized in different size fractions by centrifugation (Centrifuge 5430, Eppendorf). Prior to sizing, particles were dispersed in deionized water with HSA in a 1:1 ratio by weight. Nanoparticles (concentration 7.5-10 g/L) were ultrasonicated for 30 min in a cup-horn system (Vibracell, 90 % amplitude, 28 s ON, 2 s OFF) as described previously (Gschwend, et al., 2019). To wash out unbound HSA, the dispersion was centrifuged twice at 7830 rpm for 30 min and after each centrifugation step the supernatant was replaced with fresh water and ultrasonicated for 2 min. The rinsed particles were centrifuged at 500, 1000, 2000, 3000, and 5000 rpm where at each step the supernatant was removed and centrifuged at higher rpm.

Ion Leaching: For these experiments, 5 mg/ml of BaPOMn particles were dispersed together with equal mass of HSA in water by ultrasonication for 10 minutes (90 % amplitude, 28 s ON, 2 s OFF). Afterwards, this stock solution was mixed in triplicates in a 1:9 volumetric ratio with H_2O, PBS, NaCl 0.154 M in H_2O, and RPMI cell culture medium. The suspension were vortexed and kept under constant stirring at 37 °C for 24 hours. Afterwards, the suspensions were centrifuged for 10 minutes (10'000g) to separate the leached ions from the undissolved nanoparticles. The supernatant was then diluted in 1% HNO_3 for ICP-OES analysis (ICP-OES 5110, Agilent).

The BaPOMn nanoparticles and commercially available ICG were used for tissue experiments. The nanoparticles were dispersed in a 2 g/L particle concentration (as described

before, 1:1 wt ratio HSA, ultrasonicated 10 min, 90 % amplitude, 28 s ON, 2 s OFF) and mixed with warm agar solution (1 wt%, Sigma-Aldrich) for an end concentration of 1 g/L. The ICG was dissolved and mixed in a 0.01 g/L end concentration as it was found to show the brightest photoluminescence without photo quenching effects. The agar suspensions were injected in 2 mm thin and roughly 4 – 6 cm long capillaries for the measurements and afterwards cooled down. Photoluminescence was recorded by a visible to near-infrared camera with an InGaAs sensor (Ninox 640 VIS-SWIR, 640x512 pixels, Raptor photonics), integrated cooling (-15 °C) and an 850 nm long-pass filter. Camera height was always adjusted to record a high-resolution image and the entire illuminated area.

The performance in deep tissue imaging was compared between BaPOMn nanoparticles and commercially available ICG by stacking turkey filets of roughly 1 – 2 mm thickness on the phosphor containing capillaries. These capillaries (diameter = 2 mm, length = 4 – 6 cm) with relatively high emitter concentration (c_{BaPOMn} = 1 g/L and c_{ICG} = 0.01 g/L) were excited as previously mentioned under harmless laser conditions (808 nm, 0.19 W/m^2) and exposure times were always adjusted below the saturation point of the camera (range: 1 – 50 ms, high gain) and normalized by the maximum intensity possible afterward (16383 pixel counts). MATLAB was used to convert images to intensity profiles for further quantitative analysis of the signal-to-background ratio (SBR) and full-width half-maxima (FWHM). The SBR was evaluated dividing the sum of the 50 strongest intensities by the mean background intensity with laser exposure but without fluorescent agents at given exposure time. The FWHM was extracted by fitting three y-axis intensity profiles in the middle of the image (320 pixels) ± 30 pixels and by applying a Gauss fit.

Temperature sensing through tissue was performed within the same spectrofluorimeter system by placing tissue slices around a temperature-controlled cuvette filled with BaPOMn nanoparticles (2 g/L). The given results correspond to the average and standard deviation of

three consecutive measurements. Relative thermal sensitivity for the three cases was calculated using the following formulas (Marciniak, et al., 2016):

- Approach using the total integrated intensity:

$$S_R = \frac{1}{Intensity_{37^\circ C}} \frac{\partial Intensity}{\partial T}$$

- Approach using fluorescence intensity ratio (FIR):

$$S_R = \frac{1}{FIR_{37^\circ C}} \frac{\partial FIR}{\partial T}$$

- For the line shift, the sensitivity needs to be normalized to the spectral shift (ν , in cm^{-1}) between emission lines at T = 310 and T = 0 K, unlike the spectral line position at considered temperature.

$$S_R = \frac{1}{\nu_{0K} - \nu_{310K}} \frac{\partial \nu}{\partial T}$$

where ν_{0K} was calculated using parameters obtained from an exponential fitting:

$$\nu = \nu_0 + A \exp(R_0 T)$$

Table S1: Reported fluorescent thermometers operating in the NIR-II

Material	λ_{ex} [nm]	λ_{em} [nm]	Thermal Read-out	Rel. Sensitivity [%/K]	Authors
Ag/Ag_2S	808	1250	Intensity	~5	Ruiz et al.[46]
LaF_3:Er,Yb@Yb,Tm	690	1000/1550	Ratio	3.5	Ximendes, et al., 2017
Hybrid: (QDs + Nd^{3+})	808	1060/1250	Ratio	2.5	Ceron, et al., 2015
Ag/Ag_2S	808	1250	Ratio	2	Ruiz et al.[46]
$NaGdF_4$: Nd, Er, Ho, Yb	806	1180/1350	Ratio	1	Skripka, et al., 2017
$SrTiO_3:Ni^{2+},Er^{3+}$	375	1250	Ratio	0.76	Matuszewska et al.[22]
$YVO_4:Nd^{3+}$	808	1064	Peak Pos.	0.75 (or 0.003 nm/K)	Kolesnikov, et al., 2017a
$YVO_4:Nd^{3+}$	808	1064	Ratio	0.54	"
$YVO_4:Nd^{3+}$	808	1065/1066	Ratio	0.48	Kolesnikov, et al., 2017b
Nd@Yb core shell	790	1000-1350	Ratio	0.44	Ximendes, et al., 2016
$Y_2O_3:Nd^{3+}$	808	1053/1075	Ratio	0.43	Kolesnikov, et al., 2018
$Gd_2O_3:Nd^{3+}$	808	1350	Ratio	0.23	Balabhadra, et al., 2016
$CaF_2:Nd^{3+},Y^{3+}$	808	1053/1062	Ratio	0.18	Quintanilla, et al., 2018
$KGd(WO_4)_2:Nd^{3+}$	808	1076/1068	Ratio	0.16	Savchuk, et al., 2016
$YVO_4:Nd^{3+}$	808	1064	Ratio	0.15	Kolesnikov, et al., 2016
$YVO_4:Nd^{3+}$	808	1064	FWHM	0.14	Kolesnikov, et al., 2017a
$BiVO_4:Nd^{3+}$	750	1063/1069	Ratio	0.09	Gschwend et al.[14]
BaPOMn ($Ba_3(P_{0.99}Mn_{0.01}O_4)_{1.935}$)	808	1100-1350	Intensity	0.26	This work
"	808	1190/1250	Ratio	0.43	"
"	808	1190	Peak Pos.	0.33 (or 0.022 nm/K)	"
$Ba_5(P_{0.99}Mn_{0.01}O_4)_3OH$	808	1100-1350	Intensity	0.81	"
"	808	1170/1240	Ratio	0.34	"
"	808	1170	Peak Pos.	0.51	"

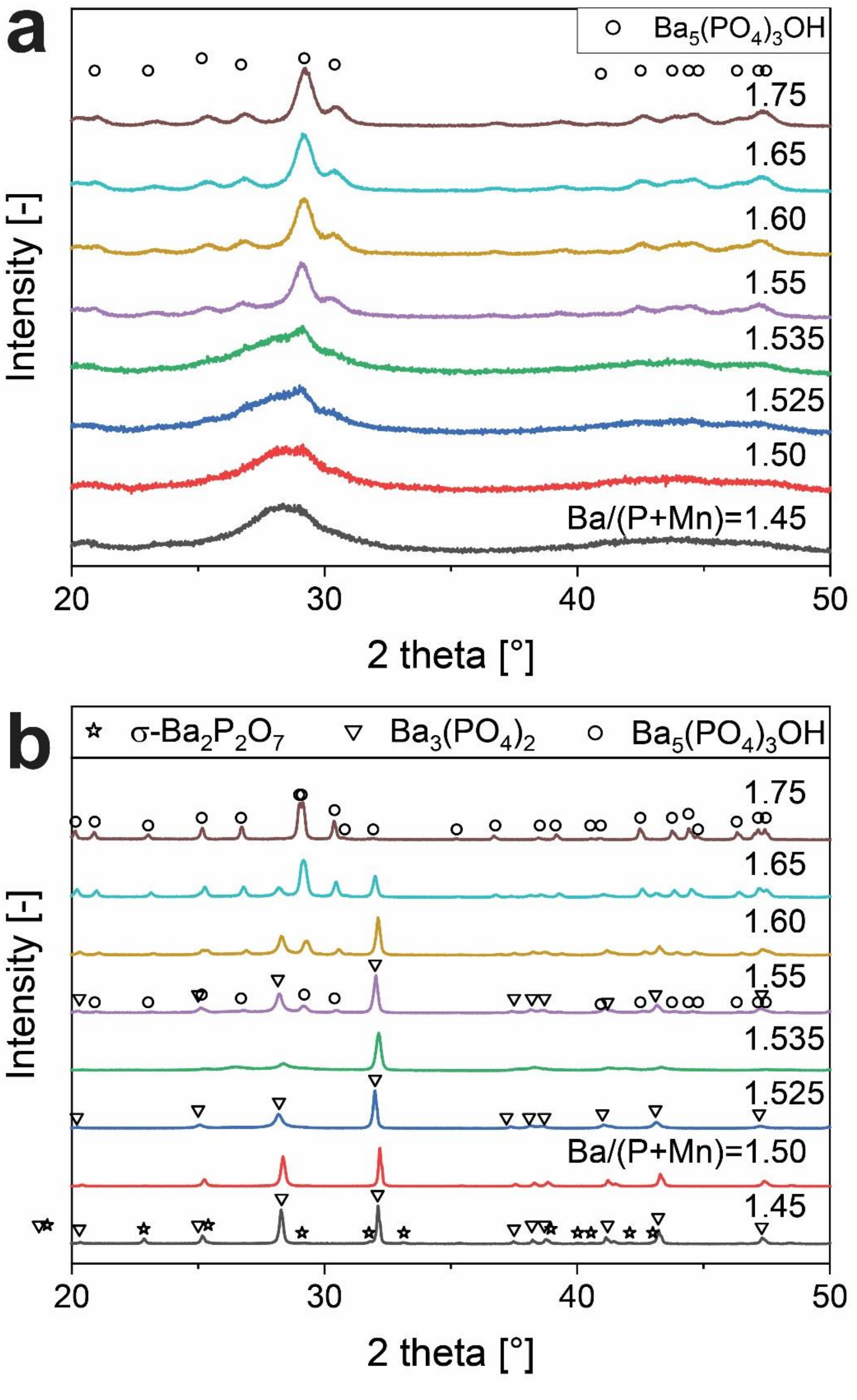

Figure S1: a) XRD patterns of as-prepared $Ba_3(P_{0.99}Mn_{0.01}O_4)_y$ particles at various Ba/(P+Mn) ratios. The particles are mostly amorphous with peaks from $Ba_5(PO_4)_3OH$ (circles, ICDS no.: 62283). **b)** XRD patterns of annealed $Ba_3(P_{0.99}Mn_{0.01}O_4)_y$ at 800 °C for 2h in air (BaPOMn). Below Ba/(P+Mn) = 1.5, both hexagonal σ-$Ba_2P_2O_7$ (stars, ICDS no.: 80101) and rhombohedral $Ba_3(PO_4)_2$ (triangles, ICDS no.: 69450) are observed. For Ba/(P+Mn) above 1.55, hexagonal $Ba_5(PO_4)_3OH$ (circles) can be detected.

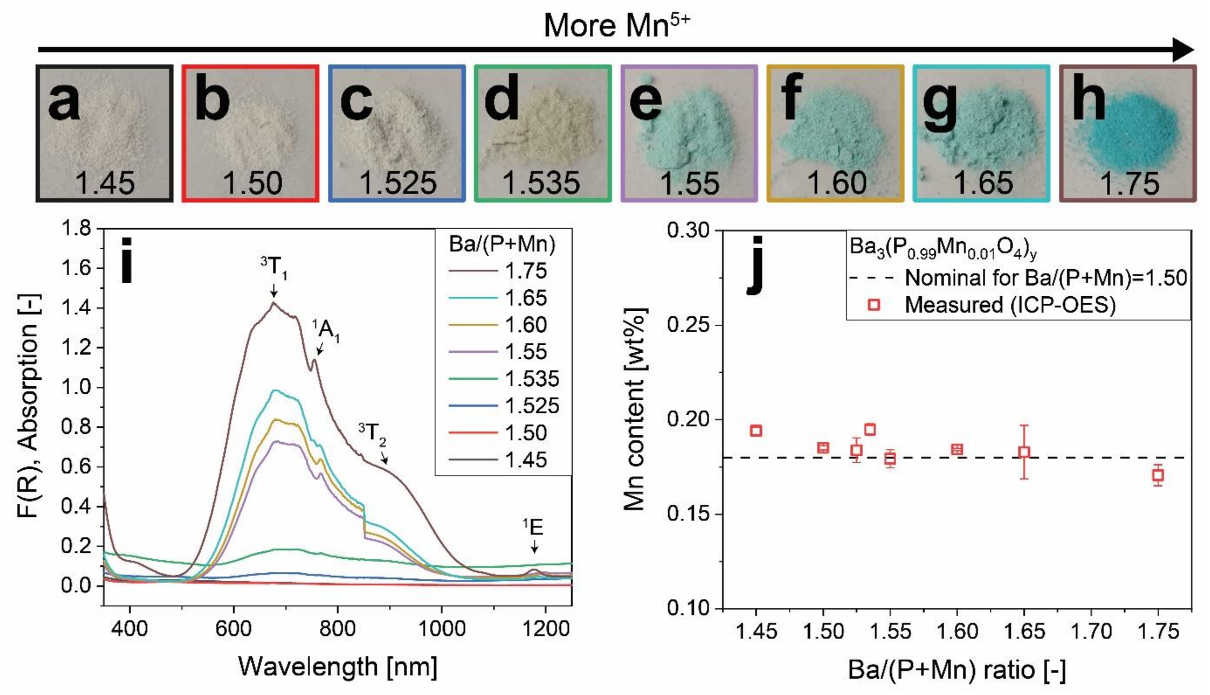

Figure S2: Effect of Ba/(P+Mn) ratio on Mn^{5+} stabilization of annealed $Ba_3(P_{0.99}Mn_{0.01})_y$ nanoparticles. **a)-h)** Powder images at various ratios **i)** absorption spectra with assignment of energy transitions from the ground $^3A_2$ to various excited states, confirming the Mn^{5+} state (Laha, et al., 2011). **j)** Measured Mn content by ICP-OES, confirming similar presence of Mn (all valence states) for all ratios.

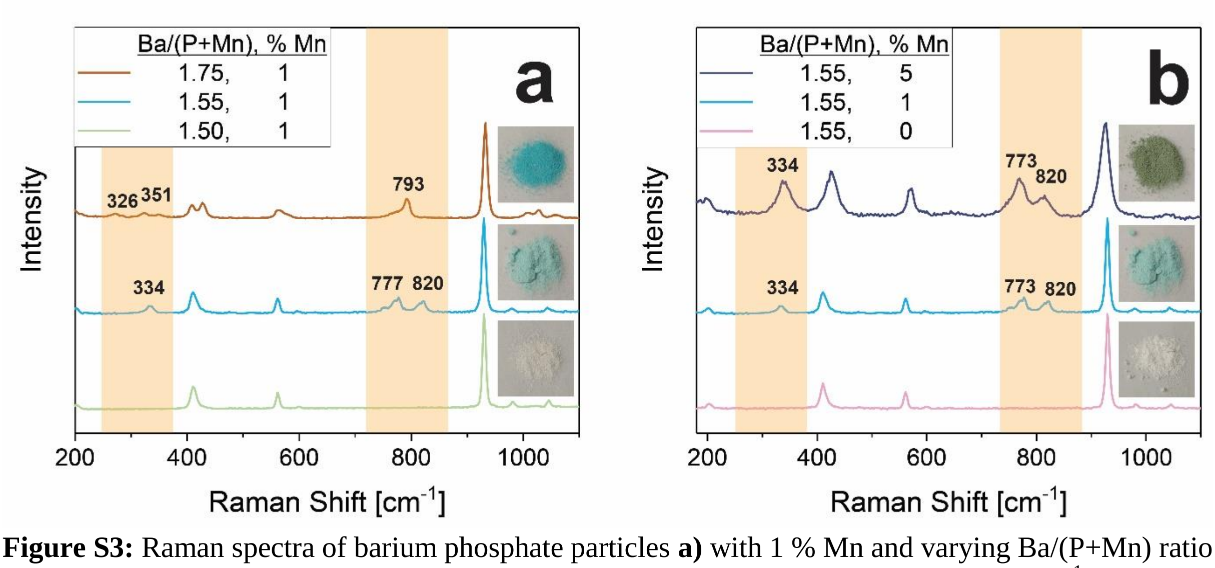

Figure S3: Raman spectra of barium phosphate particles **a)** with 1 % Mn and varying Ba/(P+Mn) ratio and **b)** varying Mn concentration and Ba/(P+Mn) = 1.55. The peaks between 770 - 820 cm^{-1} and 300 – 350 are assigned to Mn^{5+} (in $Ba_3(PO_4)_2$ (Tarte and Thelen, 1972) and in $Ba_3(PO_5)_3OH$) (Dardenne, et al., 1998), respectively in agreement with the coloration of the powder (insets).

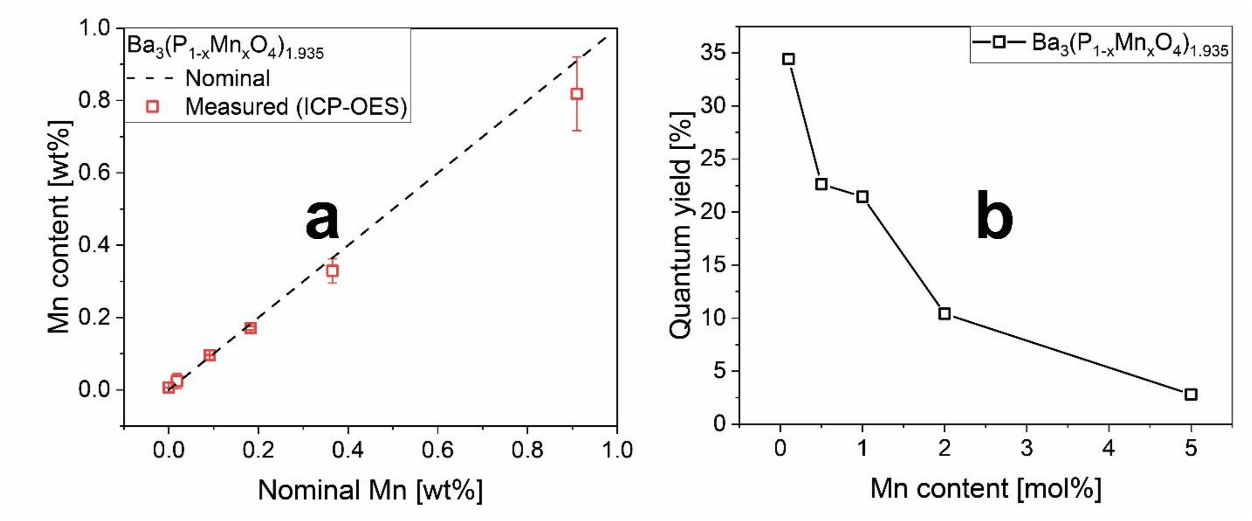

Figure S4: a) Measured Mn concentrations (0 – 5 mol %) by ICP-OES in annealed $Ba_3(P_{1-x}Mn_xO_4)_{1.935}$ (T = 900 °C) confirming a mass balance on Mn and **b)** absolute quantum yield measurements.

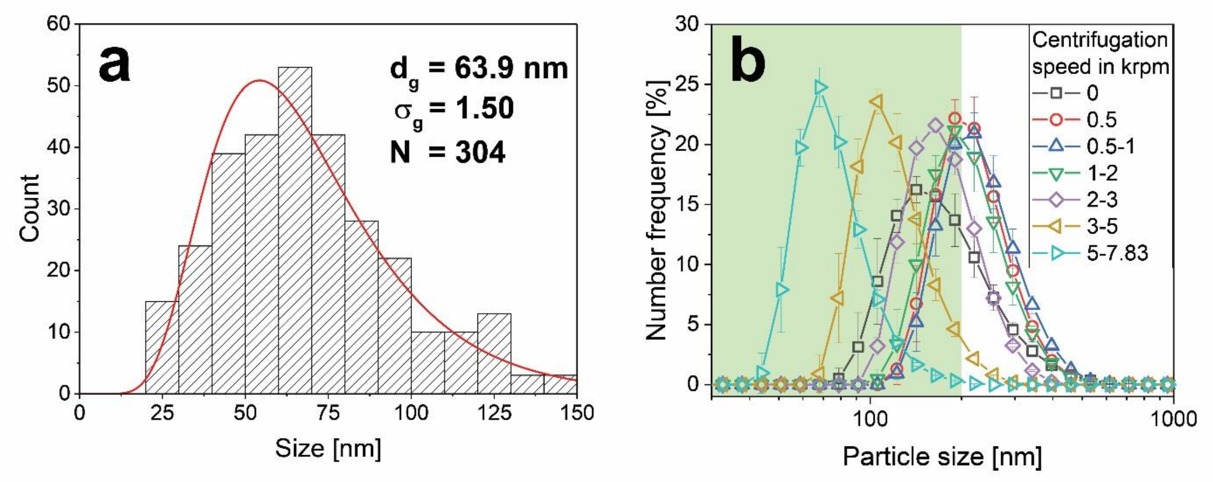

Figure S5: a) Particle size distribution of BaPOMn ($Ba_3(P_{0.99}Mn_{0.01}O_4)_{1.935}$) from TEM image analysis fitted with a log-normal distribution (red line). The geometric mean diameter (d_g) and geometric standard deviation (σ_g) are also given together with the number of counted particles (N). **b)** Number-weighted hydrodynamic particle size distributions of fractions of nanoparticle dispersions separated by centrifugation at different rates. Particles of different hydrodynamic sizes were separated by taking the supernatant after centrifugation at different speeds of aqueous dispersions of annealed BaPOMn. The shaded area corresponds to the preferred size for tumor targeting (Elsabahy and Wooley, 2012).

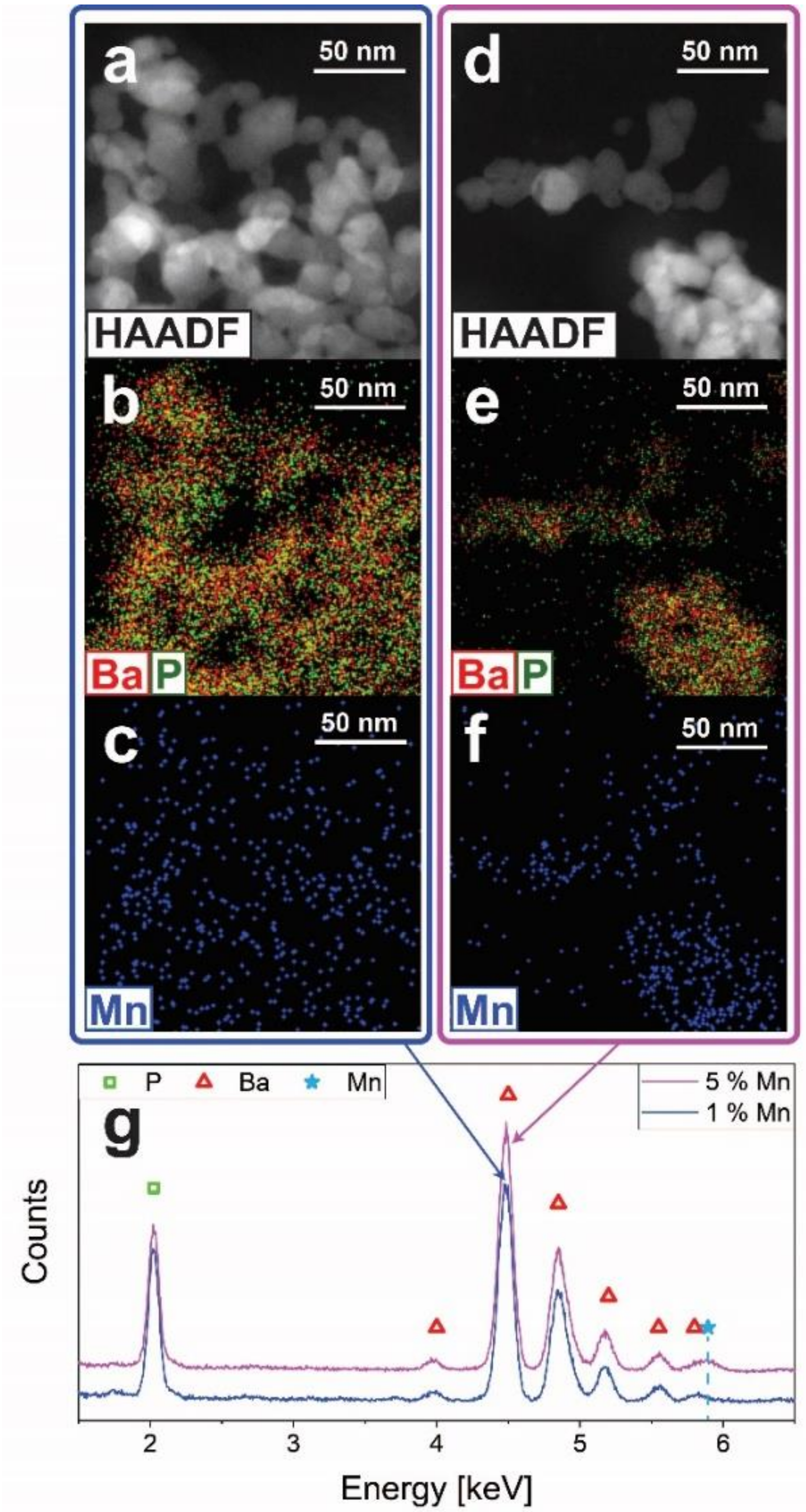

Figure S6: Electron microscopy combined with energy-dispersive X-ray analysis of $Ba_3(PO_4)_2$ doped with 1 % Mn **(a,b,c)** and 5 % Mn **(d,e,f)**. High-angle annular dark-field image **(a,d)** and corresponding barium and phosphor mappings **(b,e)**. Manganese mappings (**c,f**) need to be considered with care, as manganese (5.894 keV) is not clearly distinguishable due to its low concentration (and subsequently EDX peak intensity) and overlap with barium at 5.82 keV, as shown in the EDX spectrum **(g)**.

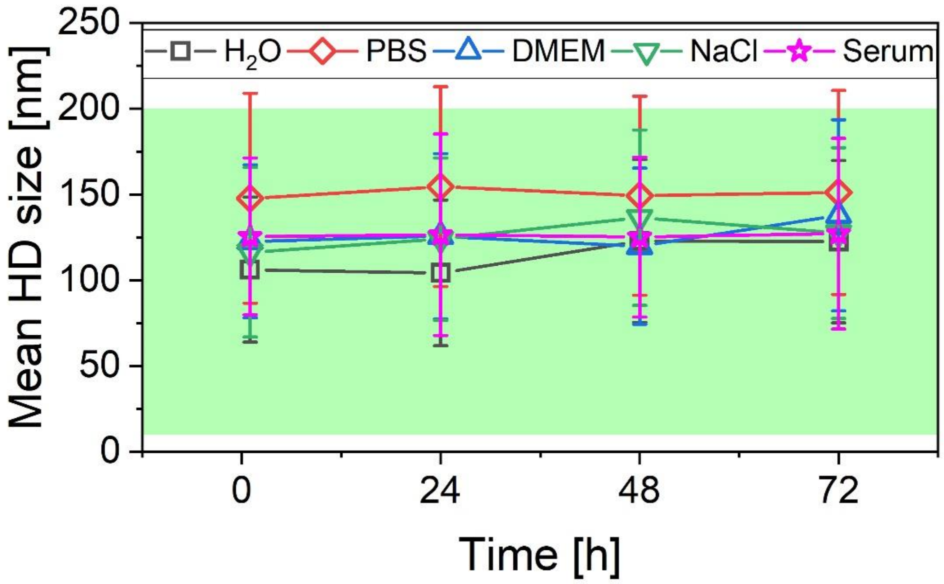

Figure S7: Colloidal stability of BaPOMn: Mean hydrodynamic size of centrifuged (supernatant of over 3000 rpm) sample from 0 to 72 h in different media (H_2O, PBS, DMEM, NaCl, serum). Green marked area corresponds to the preferred size for tumor targeting (Elsabahy and Wooley, 2012).

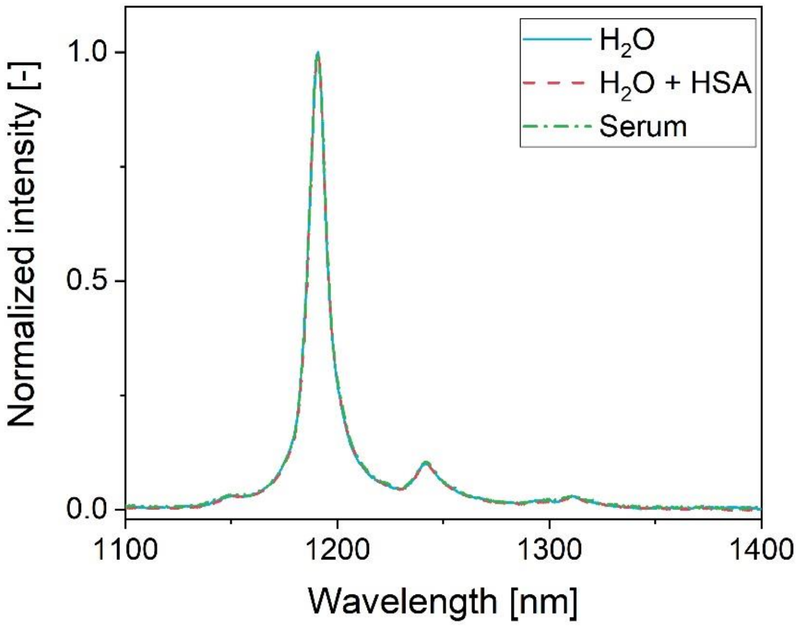

Figure S8: Emission spectra of BaPOMn particles dispersed in water before and after modification with HSA, as well as a spectrum of particles dispersed in serum.

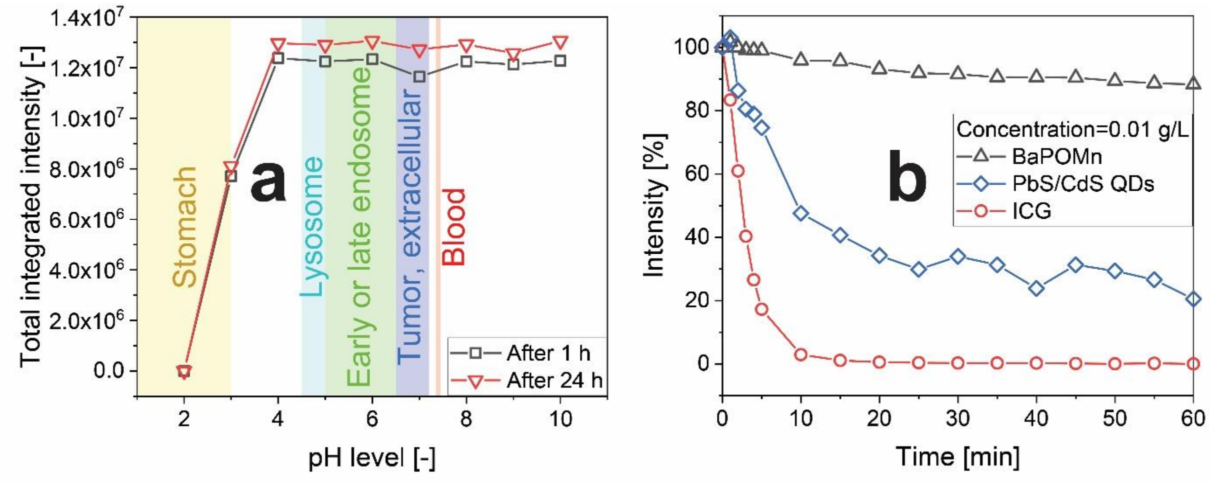

Figure S9: a) Chemical stability and fluorescence intensities at different pH with marked physiological parts after 1 (squares) and 24 h (triangles). **b)** Photostability of annealed BaPOMn, commercial ICG and PbS/CdS QDs in water with an 808 nm laser power density of 8.32 W/m^2.

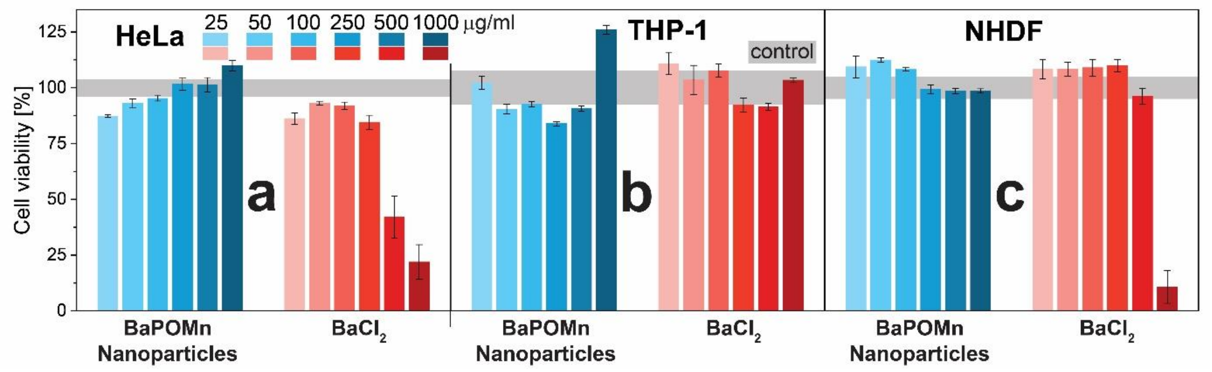

Figure S10: Reproduction of cytocompatibility testing of annealed BaPOMn nanoparticles and $BaCl_2$ salt at different concentrations of **a)** HeLa cells **b)** THP-1 monocytes **c)** NHDF cells.

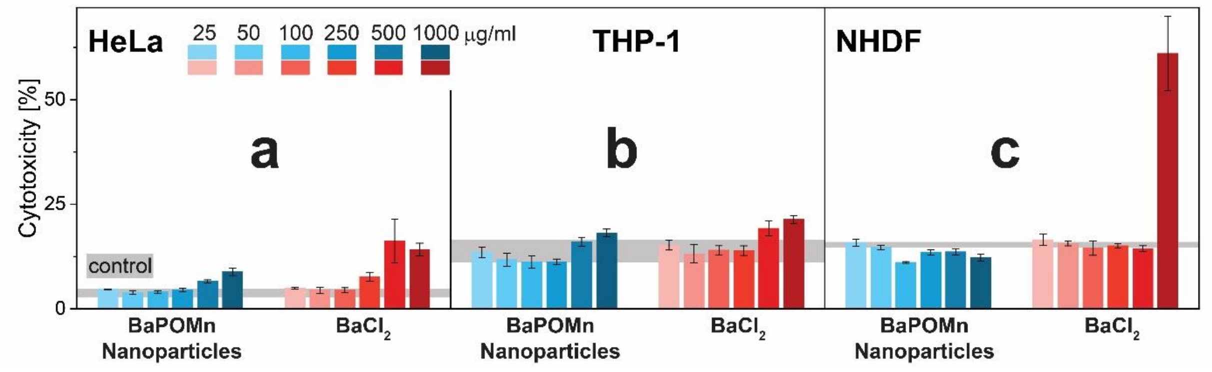

Figure S11: Cytotoxicity or lactate dehydrogenase (LDH) release of annealed BaPOMn nanoparticles and $BaCl_2$ salt at different concentrations of **a)** HeLa cells **b)** THP-1 monocytes **c)** NHDF cells. As two characteristics are measured, metabolic activity by CellTiter-Glow (Fig. 2 and S10) and membrane integrity by LDH release (Fig. S11), a linear correlation between these cannot be expected.

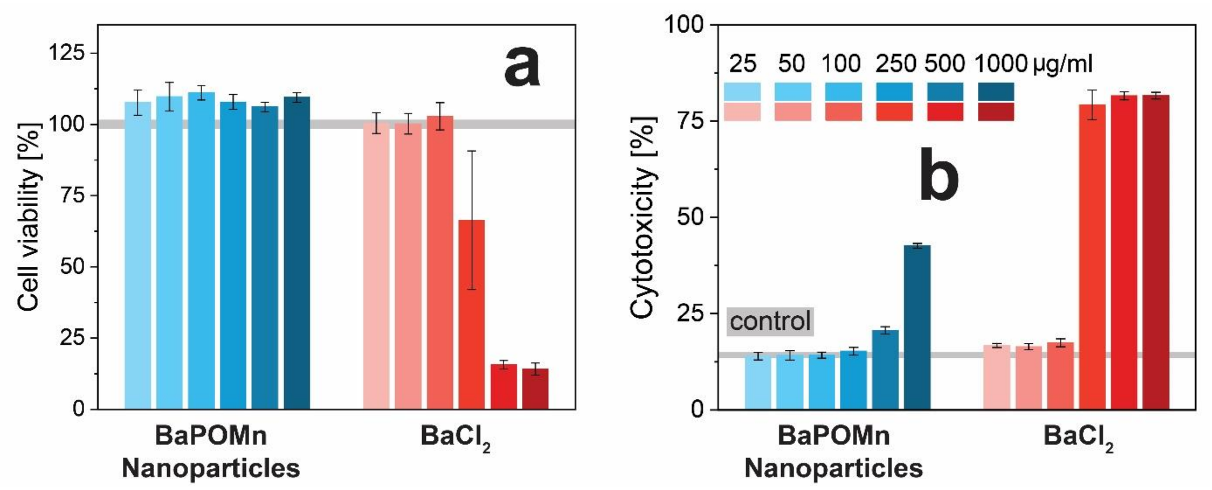

Figure S12: a) ATP activity and **b)** LDH release of HeLa cells after incubation for 48 hours with different concentrations of BaPOMn nanoparticles and $BaCl_2$ salt.

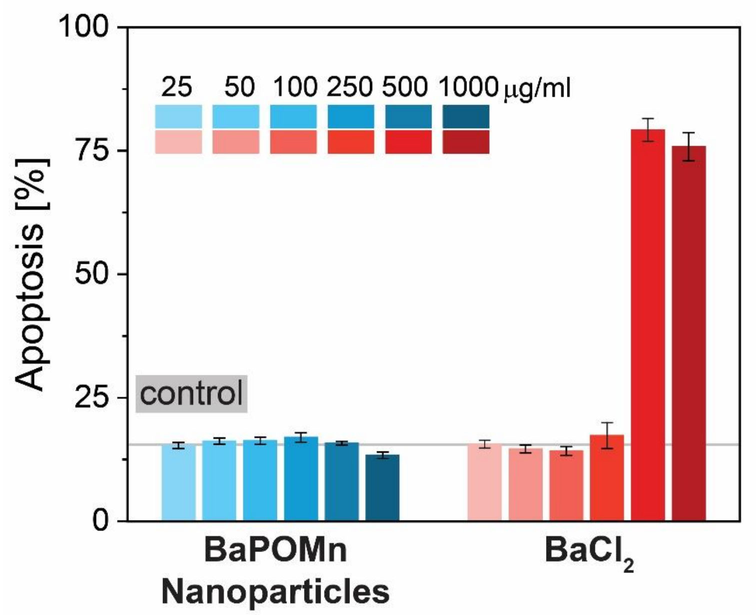

Figure S13: Apoptosis of HeLa cells after incubation with different concentration (25 – 1000 µg/ml) of BaPOMn nanoparticles and $BaCl_2$ salt.

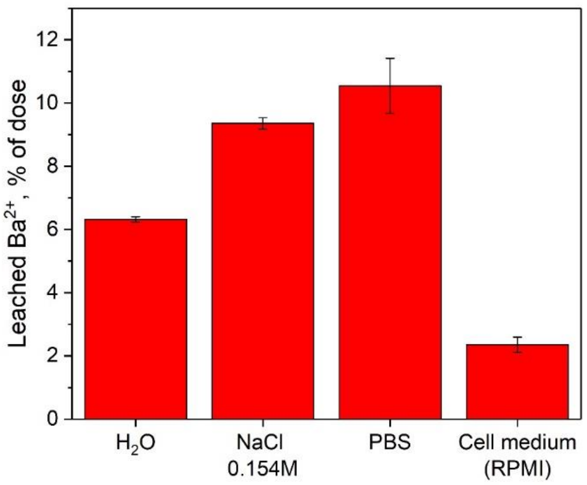

Figure S14: Leached or dissolved amount of barium from BaPOMn after 24 hours in different media, determined by ICP-OES.

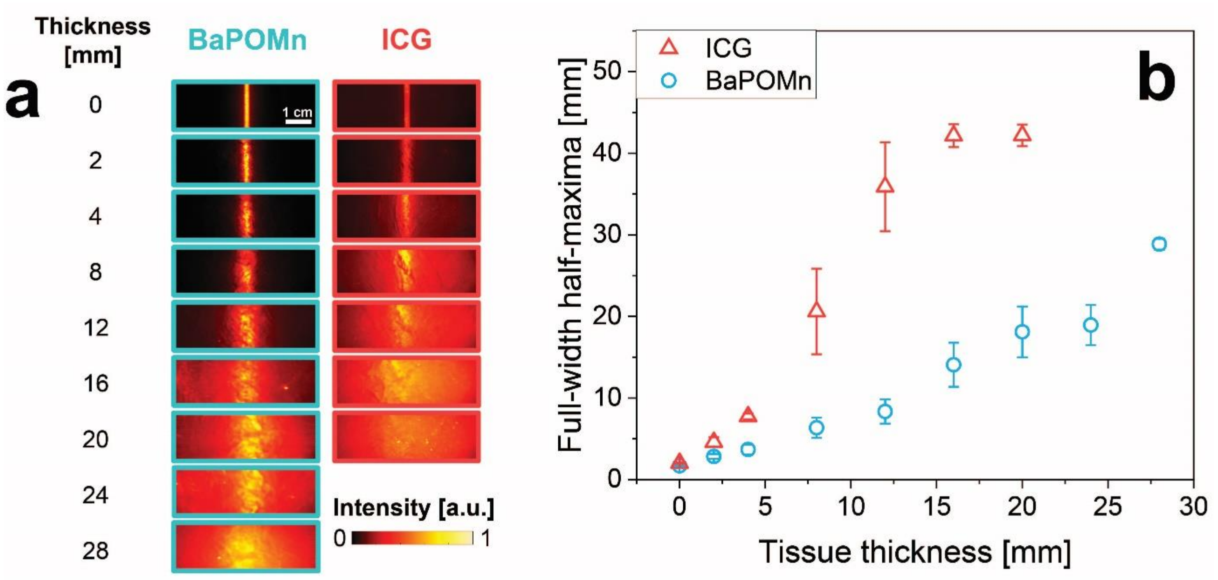

Figure S15: a) Fluorescence images of BaPOMn particles and ICG in a 2 mm capillary under animal tissue of increasing thickness. **b)** Corresponding full-width at half maximum.

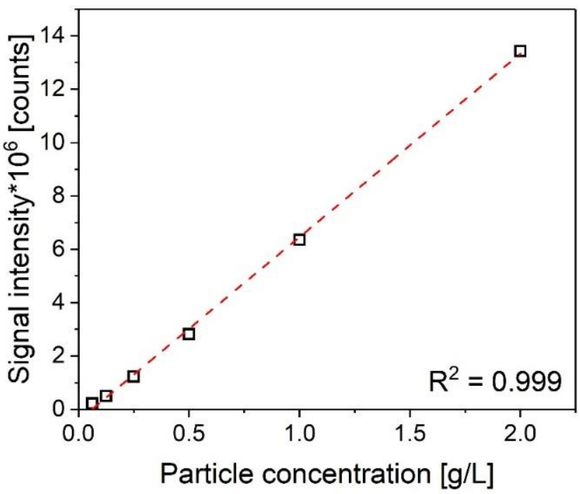

Figure S16: Integrated signal intensity of aqueous suspensions of BaPOMn particles as a function of concentration.

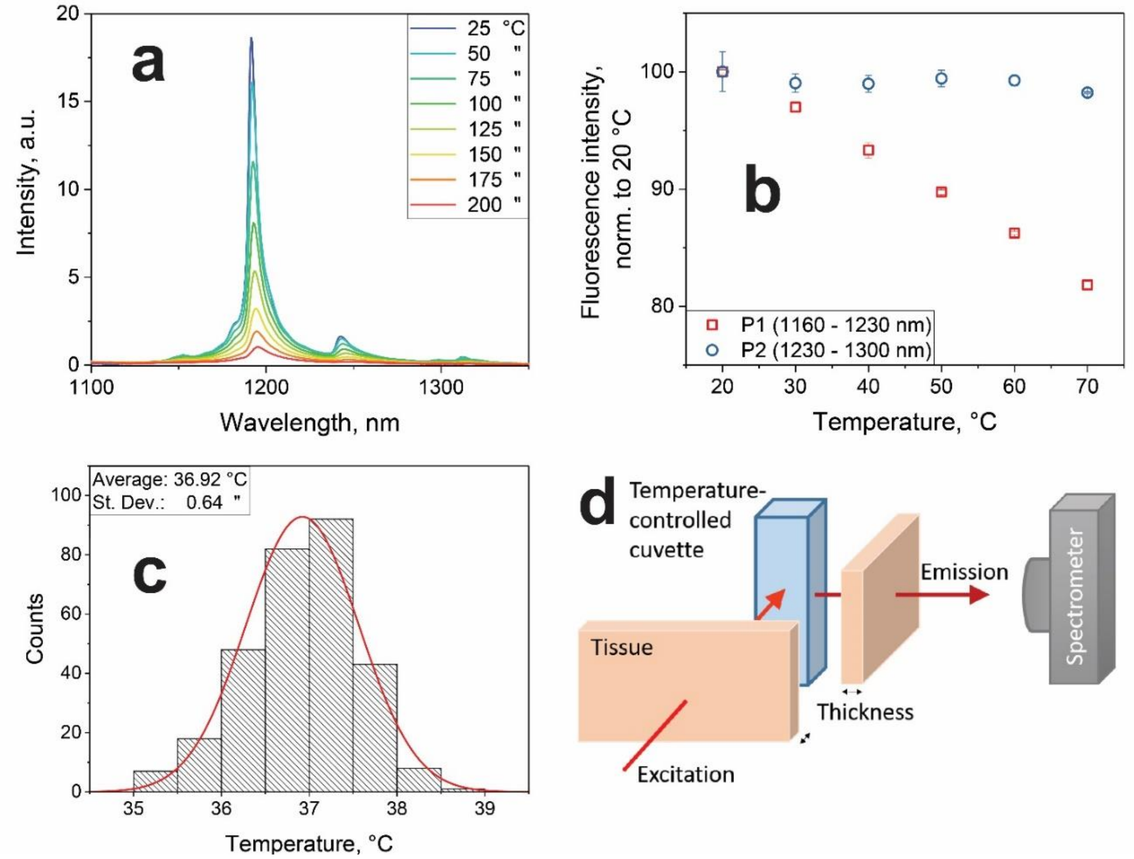

Figure S17: a) Temperature-dependent fluorescence spectra of powder between 25 and 200 °C. **b)** Thermal behaviour of the two peaks explored for ratiometric sensing (P1: 1160 – 1230 nm, P2: 1230 – 1300 nm) determined in suspension. While P1 decreases strongly with temperature, P2 remains constant up to 70 °C. **c)** Evaluated temperature distribution from 300 consecutive scans in suspension for a nominal temperature of 37 °C **d)** Sketch of the experimental setup for temperature measurements through tissue.

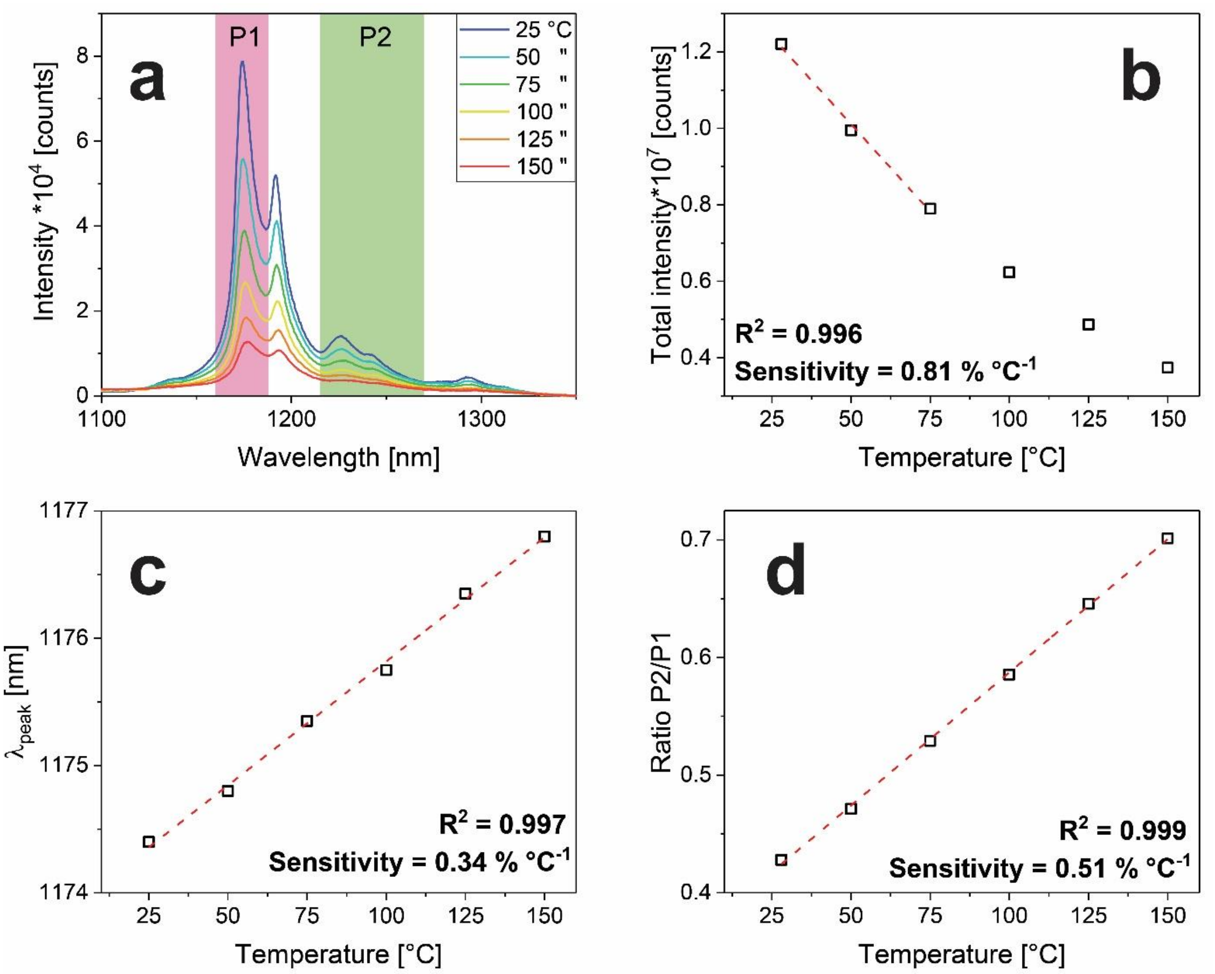

Figure S18: Thermometric performance of $Ba_5(P_{0.99}Mn_{0.01}O_4)_3OH$ powder: **a)** Temperature-dependent fluorescence spectra between 25 and 150 °C. The spectra were evaluated for thermal sensing based on **b)** total intensity, **c)** peak position and **d)** the ratio between the P1 and P2 peaks in a).

References

P. M. Gschwend, S. Conti, A. Kaech, C. Maake, S. E. Pratsinis, *ACS Appl. Mater. Inter.* **2019**, *11*, 22550.

P. Kubelka, F. Munk, *Zeitschr. für tech. Physik* **1931**, *12*, 592.

O. E. Semonin, J. C. Johnson, J. M. Luther, A. G. Midgett, A. J. Nozik, M. C. Beard, *J. Phys. Chem. Lett.* **2010**, *1*, 2445.

J. C. Boyer, F. C. J. M. van Veggel, *Nanoscale* **2010**, *2*, 1417.

L. Marciniak, A. Bednarkiewicz, D. Hreniak, W. Strek, *J. Mater. Chem. C* **2016**, *4*, 11284.

E. C. Ximendes, U. Rocha, T. O. Sales, N. Fernandez, F. Sanz-Rodriguez, I. R. Martin, C. Jacinto, D. Jaque, *Adv. Funct. Mater.* **2017**, *27*, 1702249.

E. N. Ceron, D. H. Ortgies, B. del Rosal, F. Ren, A. Benayas, F. Vetrone, D. Ma, F. Sanz-Rodriguez, J. G. Sole, D. Jaque, E. M. Rodriguez, *Adv. Mater.* **2015**, *27*, 4781.

A. Skripka, A. Benayas, R. Marin, P. Canton, E. Hemmer, F. Vetrone, *Nanoscale* **2017**, *9*, 3079.

I. E. Kolesnikov, A. A. Kalinichev, M. A. Kurochkin, E. V. Golyeva, E. Y. Kolesnikov, A. V. Kurochkin, E. Lahderanta, M. D. Mikhailov, *Sci. Rep.-Uk* **2017a**, *7*, 18002.

I. E. Kolesnikov, A. A. Kalinichev, M. A. Kurochkin, D. V. Mamonova, E. Y. Kolesnikov, A. V. Kurochkin, E. Landeranta, M. D. Mikhailov, *J. Lumin.* **2017b**, *192*, 40.

E. C. Ximendes, W. Q. Santos, U. Rocha, U. K. Kagola, F. Sanz-Rodriguez, N. Fernandez, A. D. Gouveia-Neto, D. Bravo, A. M. Domingo, B. del Rosal, C. D. S. Brites, L. D. Carlos, D. Jaque, C. Jacinto, *Nano Lett.* **2016**, *16*, 1695.
I. E. Kolesnikov, A. A. Kalinichev, M. A. Kurochkin, D. V. Mamonova, E. Y. Kolesnikov, A. V. Kurochkin, E. Landeranta, M. D. Mikhailov, *J. Lumin.* **2018**, *204*, 506.
S. Balabhadra, M. L. Debasu, C. D. S. Brites, J. Rocha, L. D. Carlos, *J. Lumin.* **2016**, *180*, 25.
M. Quintanilla, Y. Zhang, L. M. Liz-Marzan, *Chem. Mater.* **2018**, *30*, 2819.
O. Savchuk, J. J. Carvajal, L. G. De la Cruz, P. Haro-Gonzalez, M. Aguilo, F. Diaz, *J. Mater. Chem. C* **2016**, *4*, 7397.
I. E. Kolesnikov, E. V. Golyeva, M. A. Kurochkin, E. Lahderanta, M. D. Mikhailov, *Sensor Actuat. B-Chem.* **2016**, *235*, 287.
S. Laha, R. Sharma, S. V. Bhat, M. L. P. Reddy, J. Gopalakrishnan, S. Natarajan, *Bull. Mater. Sci.* **2011**, *34*, 1257.
P. Tarte, J. Thelen, *Spectrochim. Acta a-M.* **1972**, *A 28*, 5.
K. Dardenne, D. Vivien, F. Ribot, G. Chottard, D. Huguenin, *Eur. J. Solid State Inorg. Chem.* **1998**, *35*, 419.
M. Elsabahy, K. L. Wooley, *Chem. Soc. Rev.* **2012**, *41*, 2545.
